# Comparison of walking measures in patients with HTLV-1 Associated Myelopathy

**DOI:** 10.1186/1742-4690-12-S1-O17

**Published:** 2015-08-28

**Authors:** Adine Adonis, Graham P Taylor

**Affiliations:** 1 National Centre for Human Retrovirology, St Mary's Hospital, Imperial College Healthcare NHS Trust, London, UK; 2 Section of Retrovirology and GU Medicine, Department of Medicine, Imperial College, London, UK

## 

Walking, a fundamental, complex, multifunctional task, is demanding of multiple body systems, interactively affecting walking capacity. Due to their restricted walking ability, activity and participation levels in patients with HTLV-1-Associated Myelopathy (pwHAM) are compromised. Primarily, the applicability of walking endurance using the 6 minute walk (6MW) and gait speed using the timed 10m walk (10mTW), in pwHAM, were explored. Distance covered, change over one year and the influence of pain were documented. Retrospectively, case notes were abstracted for gait (6MW; 10mTW) and pain, for all pwHAM, walking a minimum of 10m, at least 11 months apart. 26 pwHAM, (8-; 18-) met the assessment criteria. Mean age 58.5 years and disease duration was 10.46years ±6.02years. Observed distance walked (55m at T1 and 71m at T2) was shorter (p<0.01) than expected for age, gender and height (610m). Using a patient's 10mTW velocity to predict the 6MW distance overestimates the actual distance walked in 6 minutes (p=.00 ;p=.00). Initially the 10mTW velocity, accounted for 35% of the variance of the 6MWdistance (F=.59; p=.00), improving to 63% (F=.00; p=.00). Patients’ 10mTW velocities versus healthy age matched controls differed at baseline (p=0.00)and follow-up (p=0.00). Using a walking aid strongly correlated with the 10mTW at both time points (rs=.78 p= .00; rs=.85 p= .00). Change in pain was not significant over time. 23%(baseline)- 42%(followup) of our pwHAM, completed the 6MW. Acting as their own controls, the distance covered for the 6MW is much shorter compared to predicted values. The 10mTW velocity underestimated the degree of disability, usefully predicted functional domains and highlighted functional decline. The 6MW, independent of the 10mTW velocity, provides a functional measure of endurance. Walking capacity in pwHAM can be measured using the 10mTW for gait speed and the 6MW for endurance. These appear to represent different indicators of walking efficacy in pwHAM.

